# Supplementary material for: Mitochondrial complex I deficiency occurs in skeletal muscle of a subgroup of individuals with Parkinson’s disease
Source: Commun Med (Lond). 2025 Apr 27;5:141. doi: 10.1038/s43856-025-00817-7 (PMC12034802; doi:10.1038/s43856-025-00817-7)
Supplement: Supplementary file 1 — Supplementary Information [file 43856_2025_817_MOESM1_ESM.pdf]

Supplementary information for

Mitochondrial complex I deficiency occurs in skeletal muscle of a subgroup of individuals with Parkinson's disease

Simon Ulvenes Kverneng *et al.*

Corresponding author: Charalampos Tzoulis

E-mail: charalampos.tzoulis@uib.no

E-mail-2: charalampos.tzoulis@helse-bergen.no

This PDF file includes:

Supplementary Figs. 1 to 13

Supplementary Tables 1 to 17

Other Supplementary Information for this manuscript include the following (separate files):

Supplementary Data 1 to 14

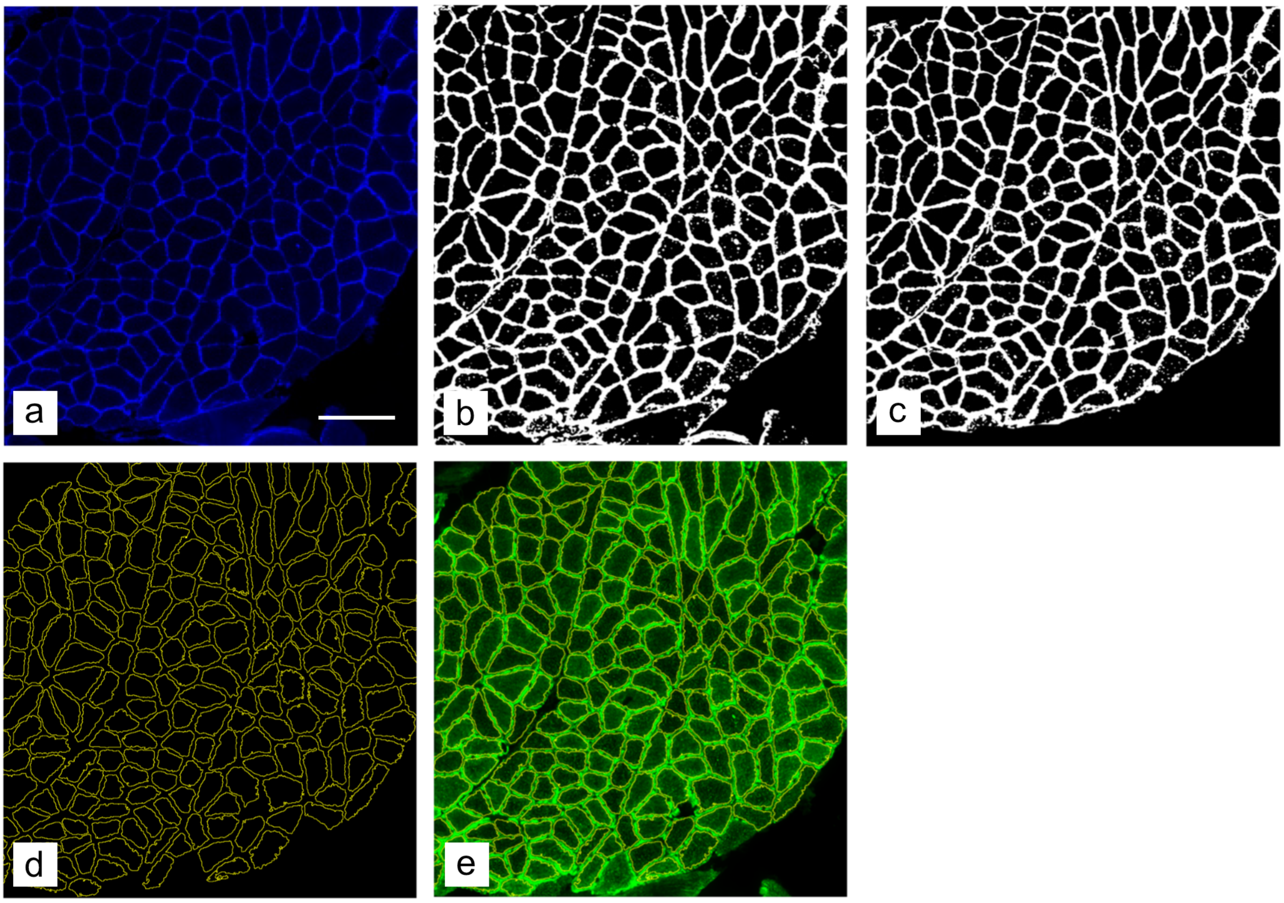

Supplementary Figure 1. Separation of individual muscle fibers based on laminin staining. **(a)** Immunohistochemistry detecting laminin. **(b)** The image is processed in ImageJ2 (version 2.3.0/1.53f) to enhance edges and blurring is added to make lines meet. The image is then converted to 8-bit and a threshold is applied and manually adjusted to make a suitable mask. **(c)** Holes in the mask are manually closed using the pencil tool (examples indicated by red arrows). **(d)** Individual regions of interest (ROIs) for each muscle fiber are created. **(e)** ROIs are used to measure fluorescence signal of VDAC1, NDUFB10 and MTCO1 for individual muscle fibers (VDAC1-signal is shown in the image as an example). Scale bar: 200 µm.

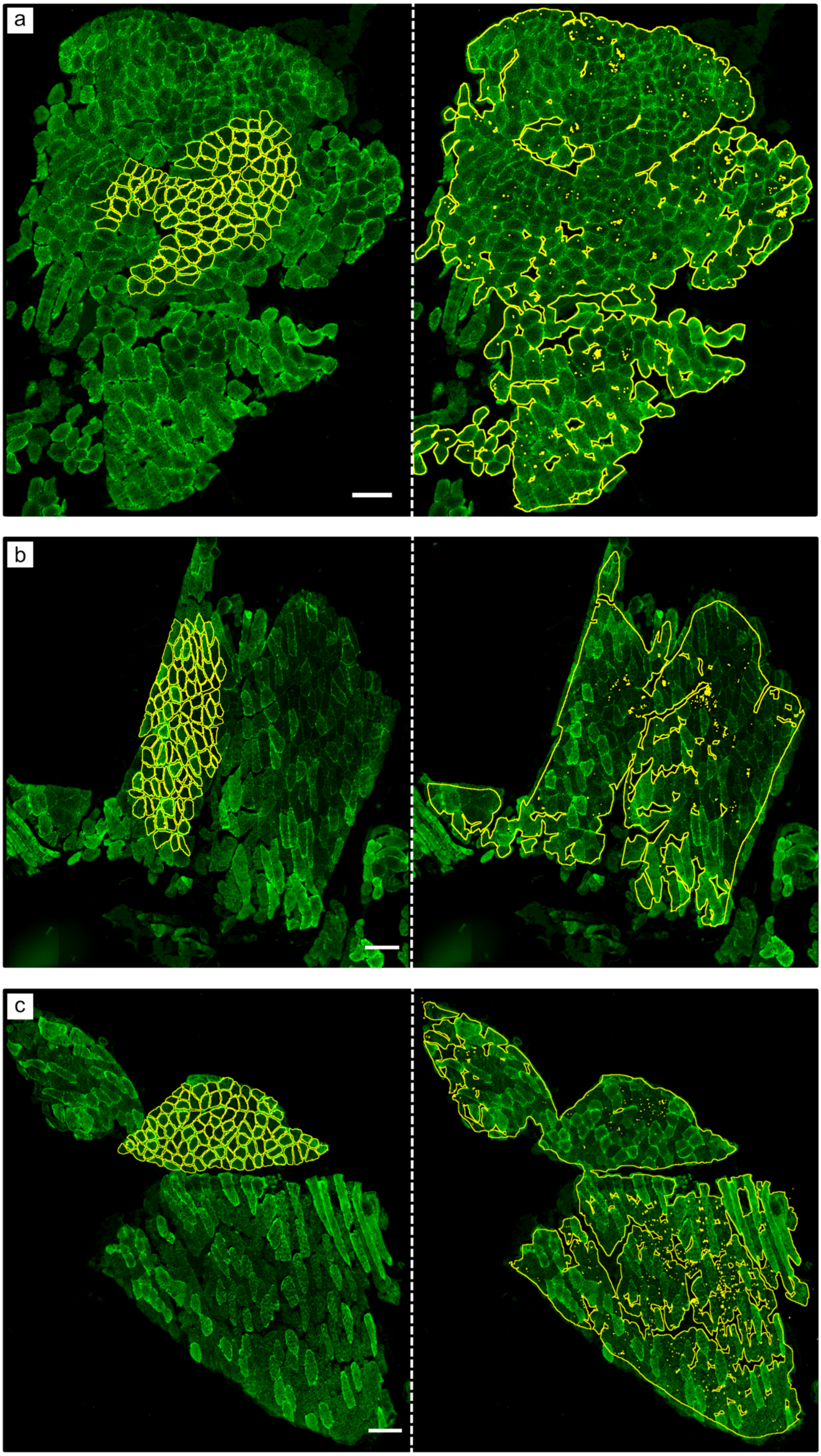
a
b
c

Supplementary Figure 2. Measurement of immunohistochemistry fluorescence signal in individual muscle fibers and large section areas.

(a-c) Three representative examples of regions of interest (ROIs) used for measuring immunohistochemistry fluorescence signal in individual muscle fibers (left images) and large section areas encompassing the majority of muscle fibers (right images) from the same sections. ROIs are indicated by yellow borders. Scale bar: 200 μm.

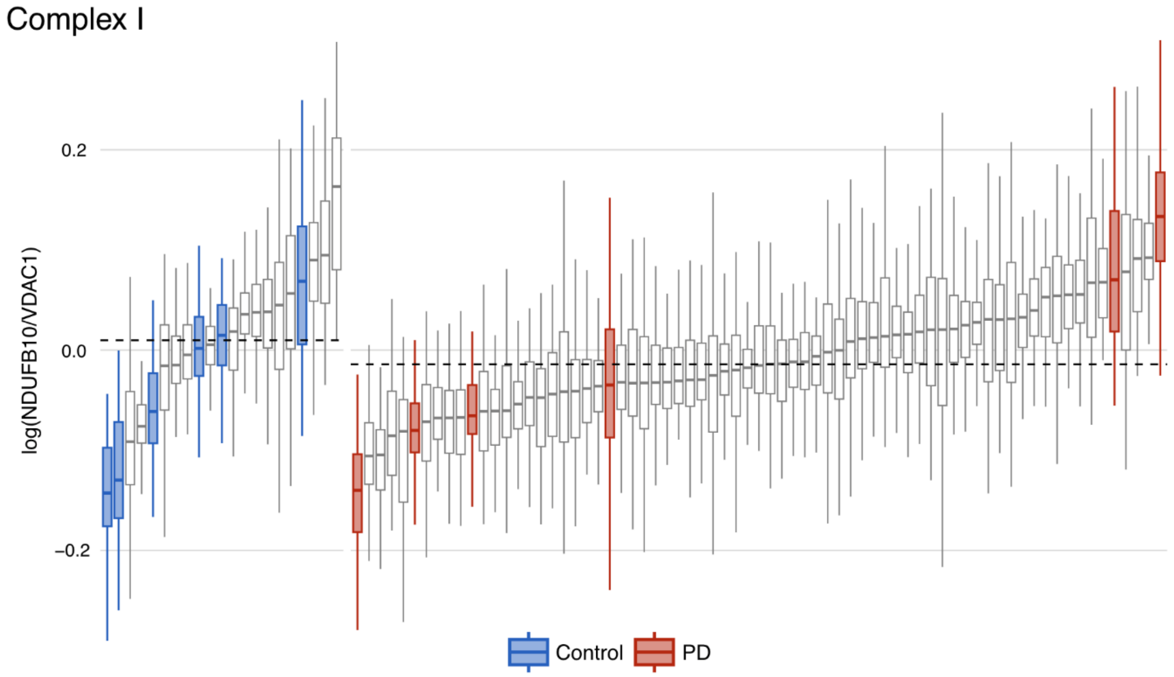

Supplementary Figure 3. Selection of samples for mtDNA analysis in single muscle fibers. Six PD samples (red) and six control samples (blue), spanning the range of median complex I quantity, were selected for laser-microdissection of multiple single muscle fibers for mtDNA analysis. Boxplots show complex I (NDUFB10) fluorescence intensity normalized to mitochondrial mass (VDAC1) in single muscle fibers (n = 75-100 per individual) in the patient and control group. Each boxplot represents one individual. Boxes: median and interquartile range (IQR); whiskers: 1.5 x IQR from the lower and upper quartiles. Individuals are sorted by median values from left to right. Dashed lines represent group-level medians of the entire PD (n = 71) and control (n = 21) group. Values are log transformed. For the purpose of visualization, the data have been adjusted for the effect of immunohistochemistry staining batch by regressing out this variable (see Methods section).

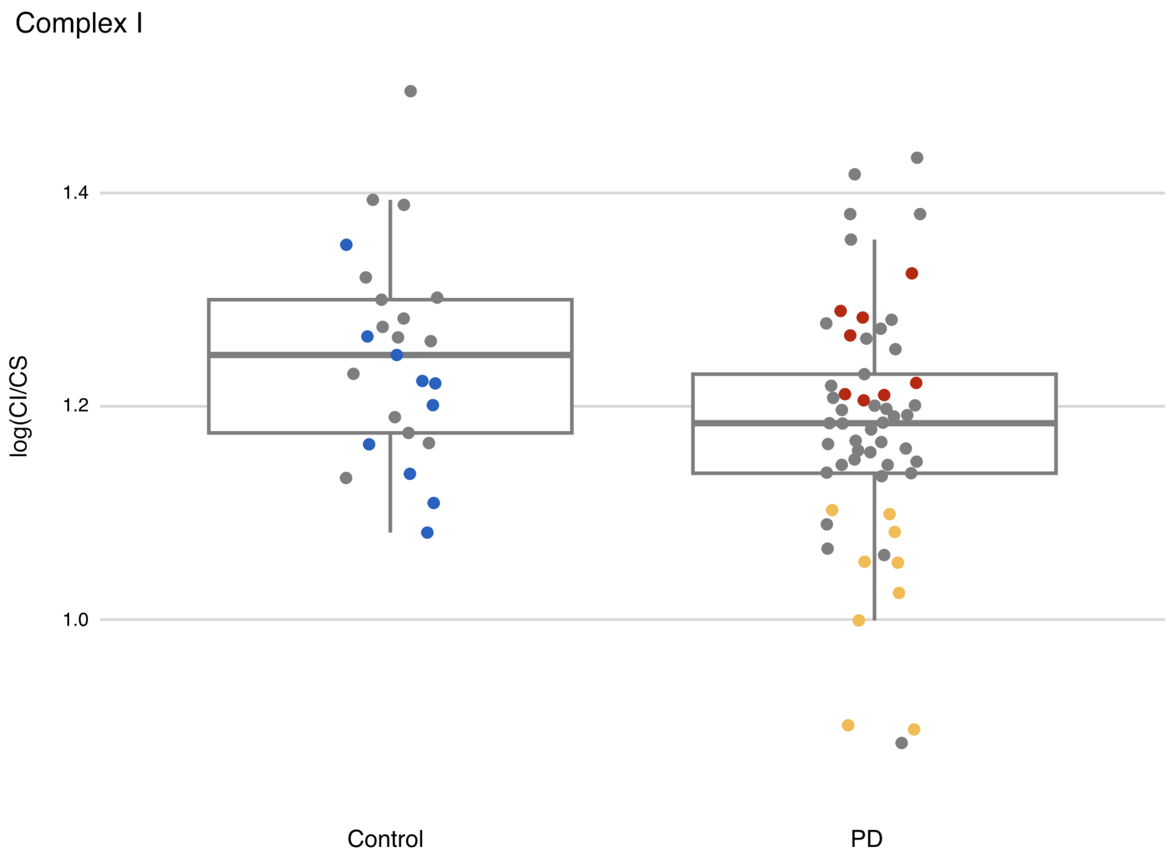

Supplementary Figure 4. Selection of samples for mtDNA analysis in bulk muscle tissue. Spectrophotometric activity measurements of complex I (CI) normalized to citrate synthase (CS) in muscle biopsies from individuals with PD and controls are shown. Smokers are not included. 8 PD samples with CS normalized CI activity similar to controls (red), 9 PD samples with low activity levels (yellow), and 10 control samples (blue) were selected for mtDNA analysis in bulk muscle tissue. The three groups were matched for age (Supplementary Table 1). Values are log transformed. The data have been adjusted for the effect of measurement batch by regressing out this variable (see Methods section). Boxes: median and interquartile range (IQR); whiskers: 1.5 x IQR from the lower and upper quartiles. Each dot represents one individual.

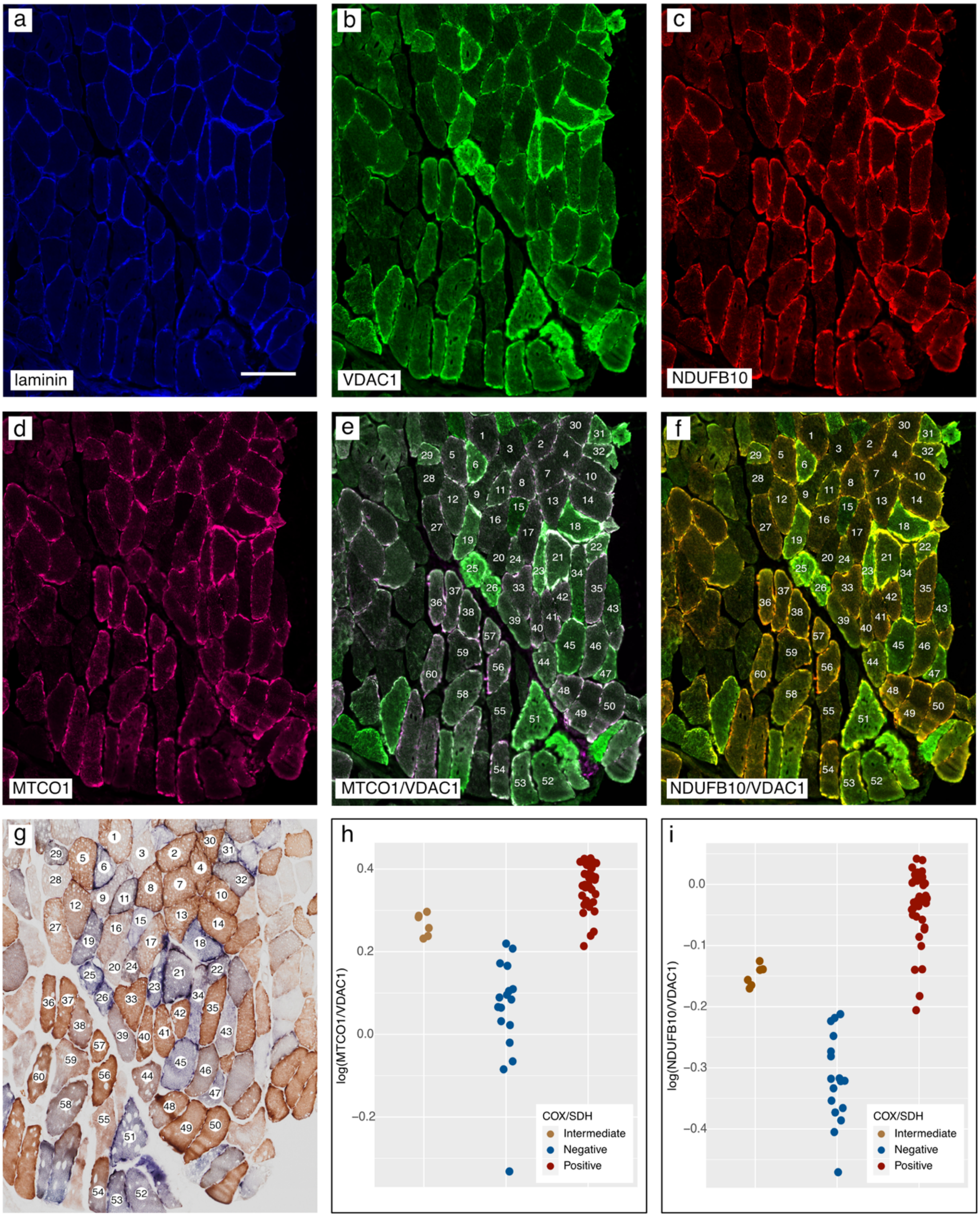
a
laminin
b
VDAC1
c
NDUFB10
d
MTCO1
e
MTCO1/VDAC1
f
NDUFB10/VDAC1
g
h
log(MTCO1/VDAC1)
0.4
0.2
0.0
−0.2
COX/SDH
Intermediate
Negative
Positive
i
log(NDUFB10/VDAC1)
0.0
−0.1
−0.2
−0.3
−0.4
COX/SDH
Intermediate
Negative
Positive

Supplementary Figure 5. Validation of quadruple immunohistochemistry for quantitative MRC complex assessment.

Quadruple immunohistochemistry and cytochrome c oxidase/succinate dehydrogenase (COX/SDH) histochemical staining of two consecutive sections from a skeletal muscle biopsy harboring the common ~5 kb single mtDNA major arc deletion. Immunohistochemistry staining of laminin **(a)**, scale bar: 200 µm, the mitochondrial marker porin (VDAC1;**b**), subunit NDUFB10 of complex I (CI; **c**), subunit MTCO1 of complex IV (CIV, **d**). **(e)** Merged MTCO1 and VDAC1 signal. **(f)** Merged NDUFB10 and VDAC1 signal. **(g)** Consecutive section from the same skeletal muscle as in a-f stained with COX/SDH histochemistry. COX-status was qualitatively assessed ("positive", "intermediate" or "negative") by two readers (SUK and CT). 60 corresponding muscle fibers are labeled in e, f and g. **(h)** VDAC1-normalized CIV fluorescence intensity (log(MTCO1/VDAC1)) compared to COX/SDH status in the 60 labeled muscle fibers. **(i)** VDAC1-normalised CI fluorescence intensity (log(NDUFB10/VDAC1)) compared to COX/SDH status in the 60 labeled muscle fibers. All fluorescence intensities were adjusted for background fluorescence.

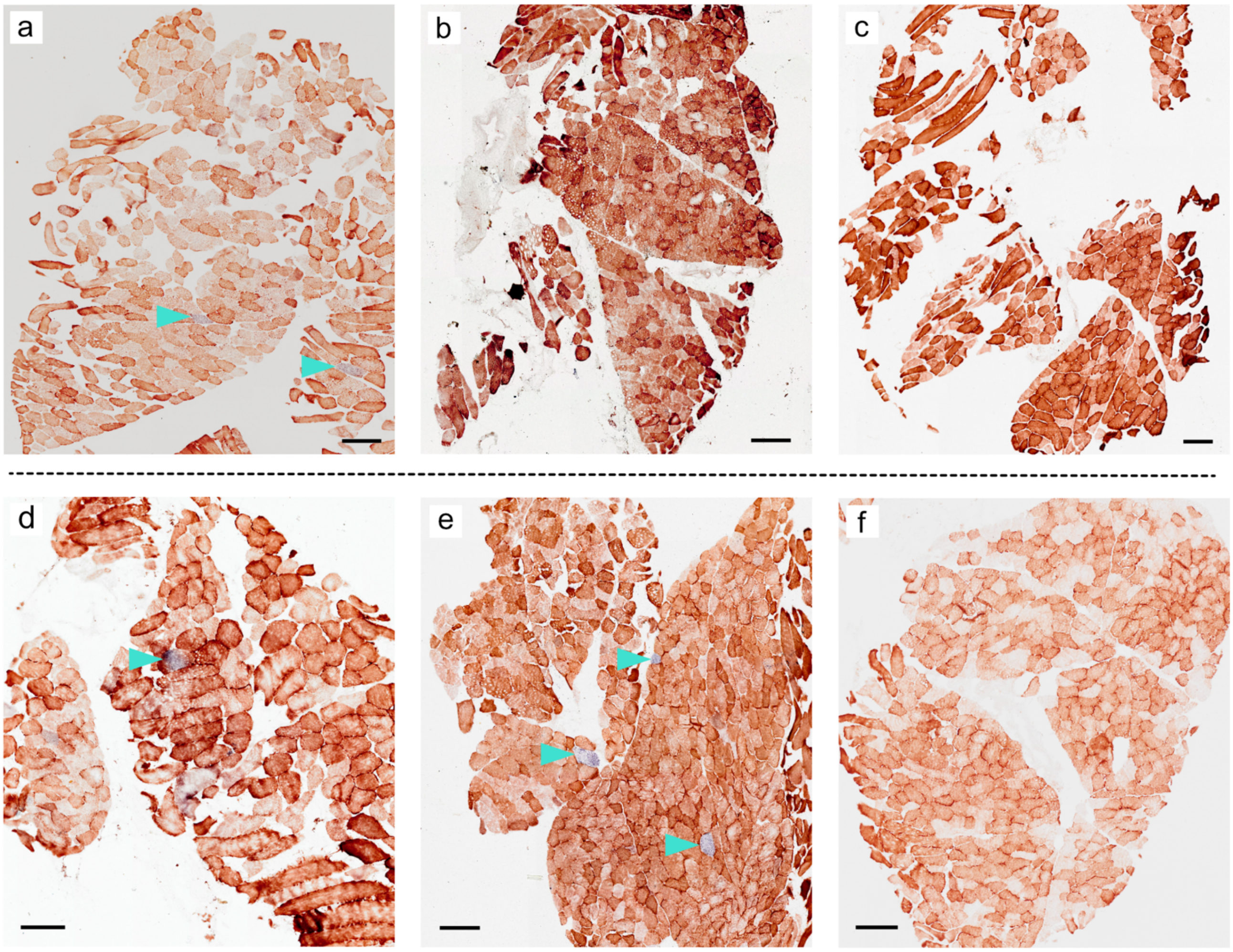

Supplementary Figure 6. Cytochrome c oxidase/succinate dehydrogenase histochemistry. Representative examples of cytochrome c oxidase/succinate dehydrogenase (COX/SDH) histochemical staining from muscle biopsies of three neurologically healthy controls (a – c) and three individuals with Parkinson's disease (d – f). COX-positive fibers stain brown, while COX-negative and SDH-positive fibers stain blue (teal arrow heads). Scale bar: 200 µm.

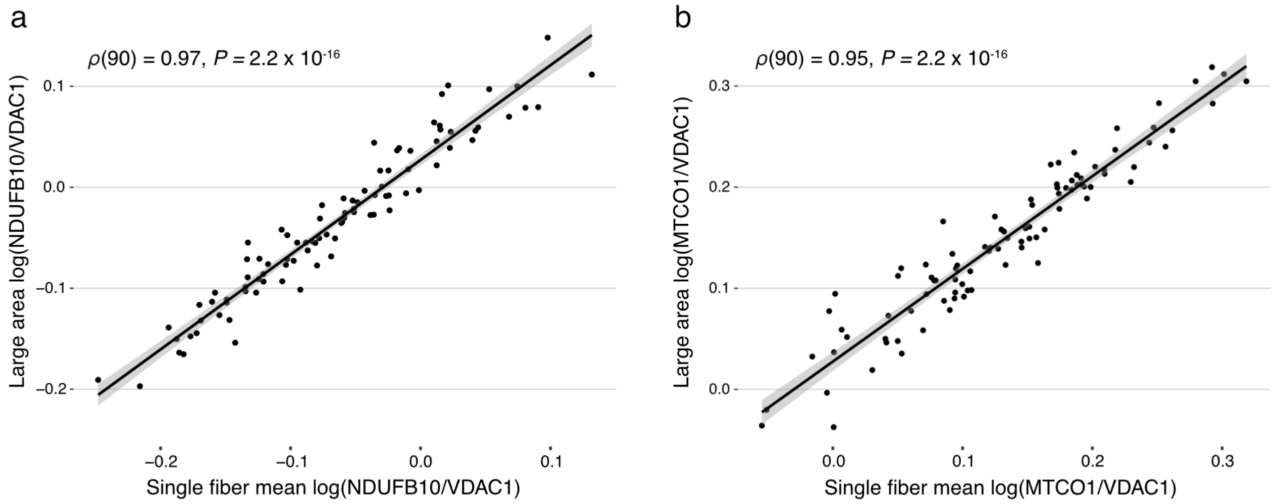

Supplementary Figure 7. Correlation between immunohistochemistry fluorescence measurement in multiple single fibers and in a large section area.

In each section, fluorescence intensity was measured in multiple single muscle fibers (n = 75 - 100 per section) and in a single large area covering the majority of fibers of the section. **(a)** Correlation between the mean of single fiber CI levels and CI level in a single large area from the same section. Individuals with PD and control individuals are plotted together. Each dot represents one individual. **(b)** Correlation between the mean of single fiber CIV levels and CIV level in a single large area from the same section. Individuals with PD and control individuals are plotted together. Each dot represents one individual.

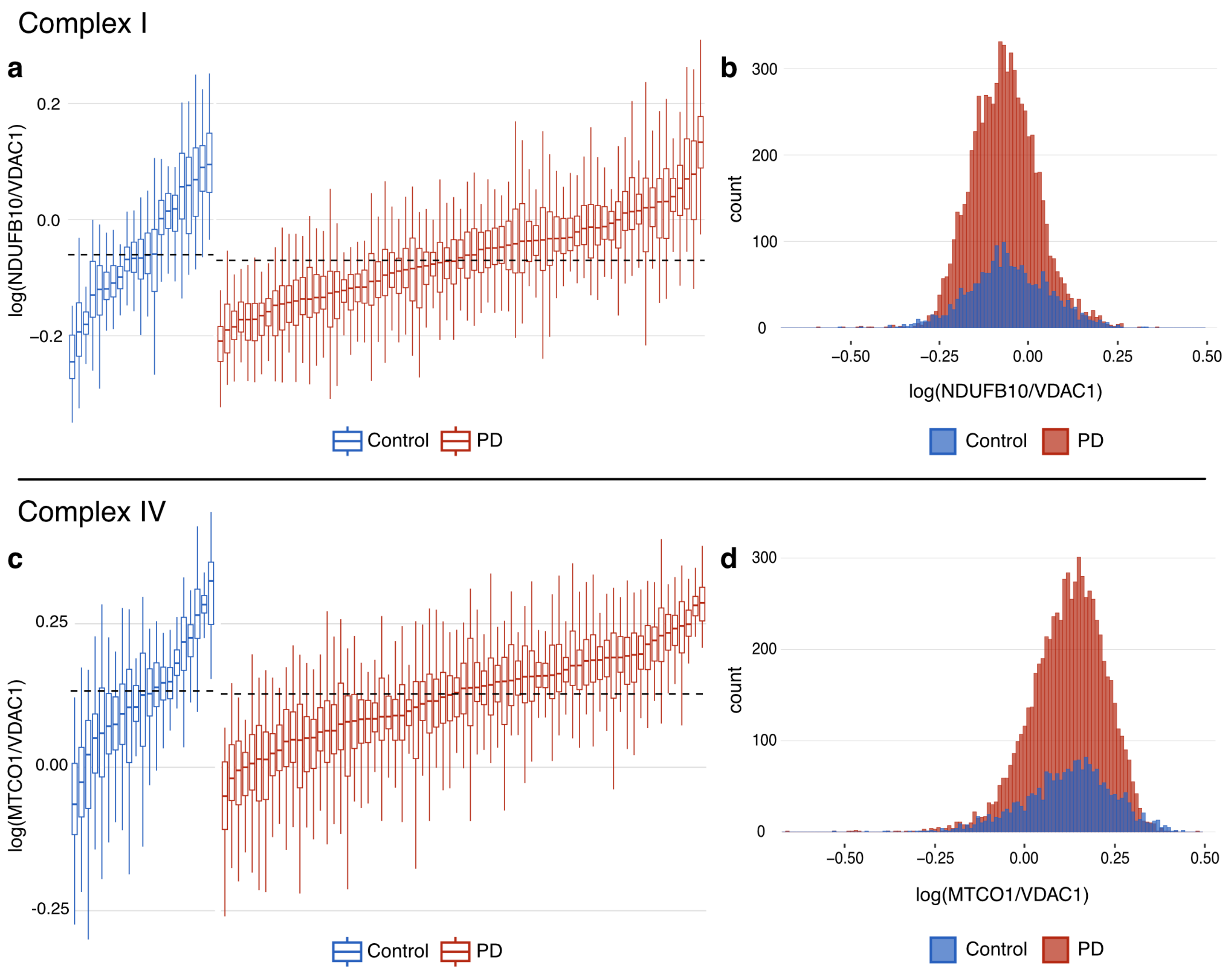

Supplementary Figure 8. Immunohistochemistry of complexes I and IV in PD single muscle fibers without adjusting for staining batch.

Complex I (NDUFB10) and complex IV (MTCO1) fluorescence intensity normalized to mitochondrial mass (VDAC1) in single muscle fibers (*n* = 75-100 per individual) in the PD (red) and control (blue) groups. Values are log transformed. Data have not been adjusted for staining batch. Boxplots **(a, c)** show individual-level distributions of single fiber measurement where each box represents one individual. Boxes: median and interquartile range (IQR); whiskers: 1.5 x IQR from the lower and upper quartiles. Individuals are sorted by median values from left to right. Dashed lines show the group-level medians of the PD and control groups. The histograms **(b, d)** represent group-level distributions of single fiber measurement in the PD and control groups.

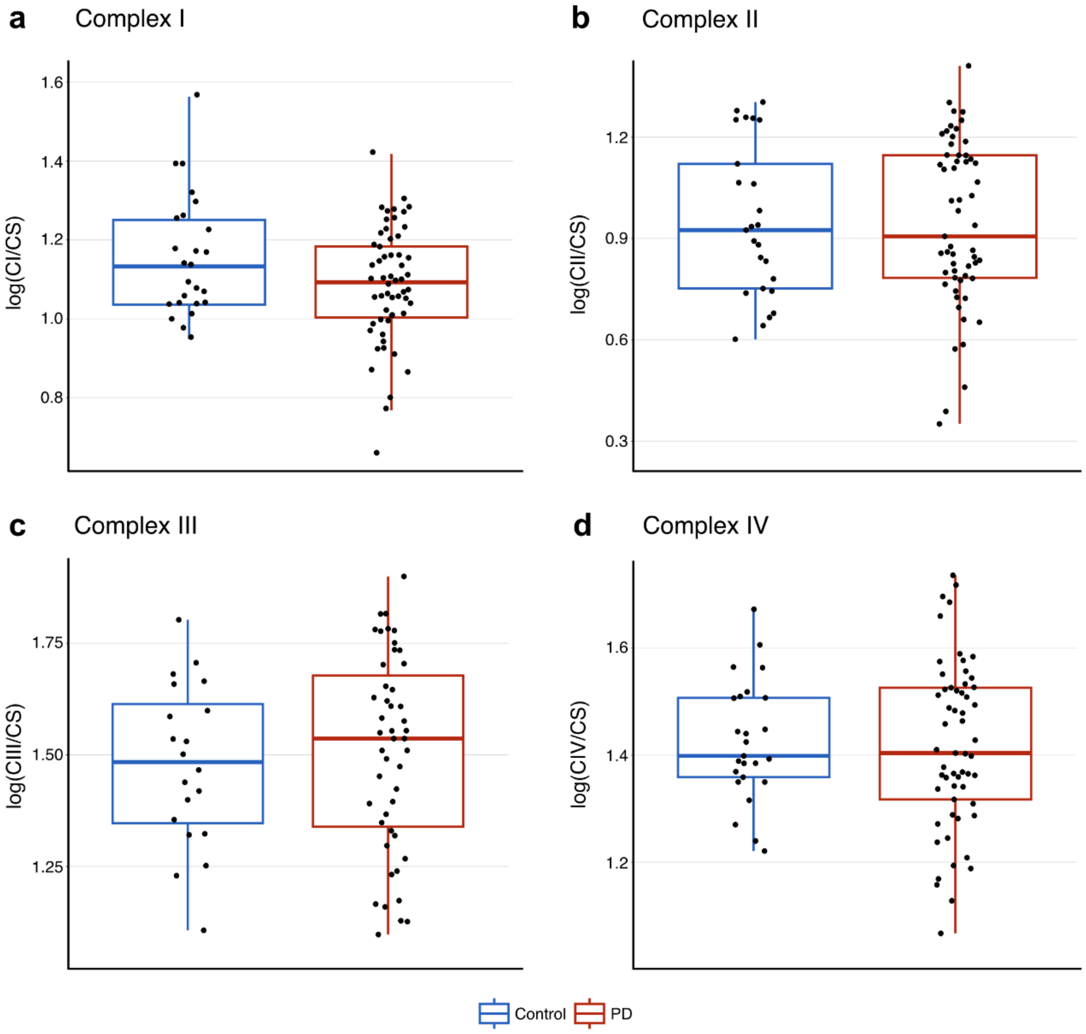

Supplementary Figure 9. Spectrophotometric activity measurement of mitochondrial respiratory complexes in muscle without adjusting for batch effects.

Activities of complexes I-IV (CI-CIV), normalized to citrate synthase (CS) activity. Values are log transformed. Smokers have been removed. Red boxplots represent the PD group, and blue boxplots represent the control group. Boxes: median and interquartile range (IQR); whiskers: 1.5 x IQR from the lower and upper quartiles. Each dot represents one individual. **(a)** CS-normalized CI activity. **(b)** CS-normalized CII activity. **(c)** CS-normalized CIII activity. Measurements from 15 samples were excluded due to technical issues with the reduction of decylubiquinone (Supplementary data 1). **(d)** CS-normalized CIV activity.

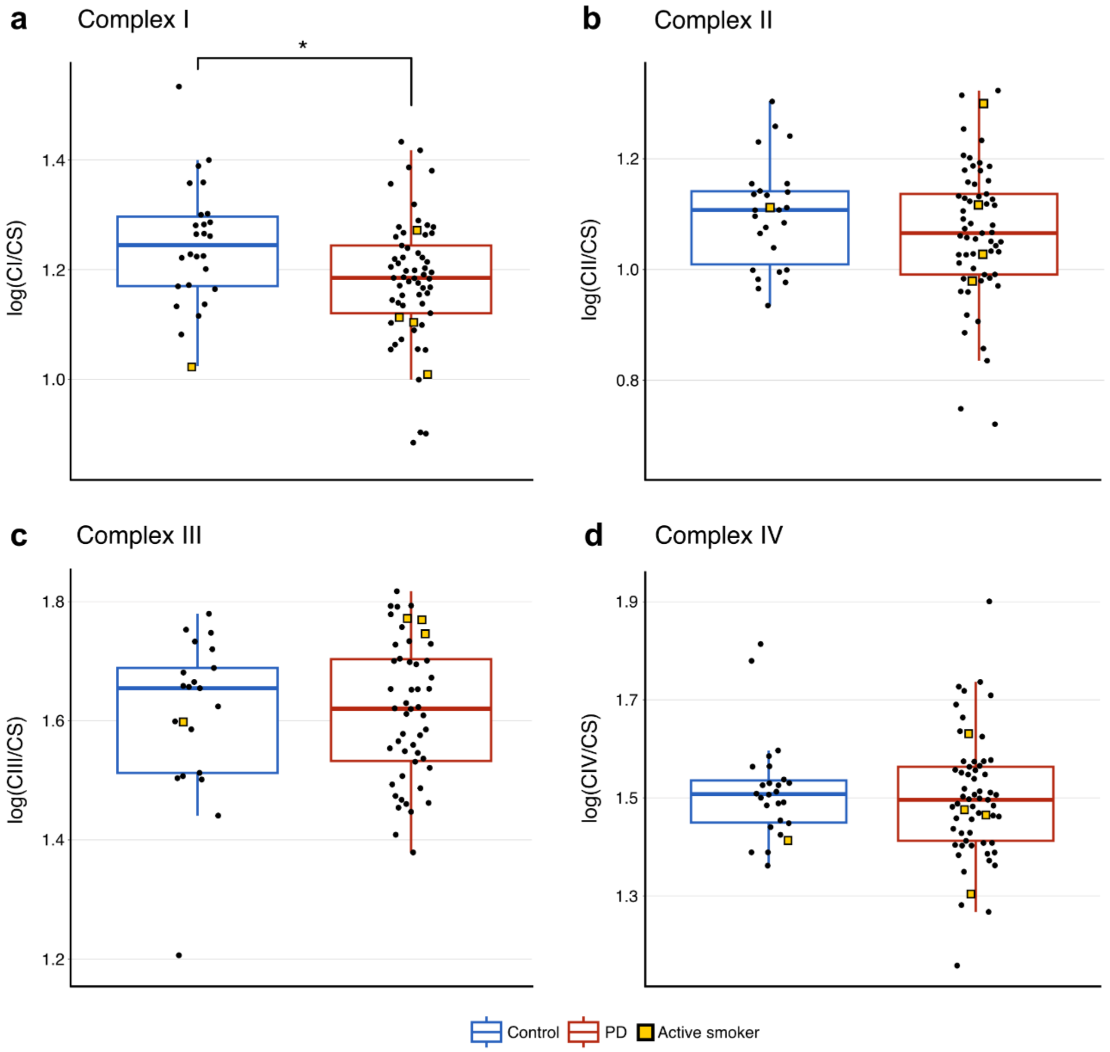

Supplementary Figure 10. Spectrophotometric activity measurement of mitochondrial respiratory complexes in muscle, including smokers and adjusting for batch effects. Activities of complexes I-IV (CI-CIV), normalized to citrate synthase (CS) activity. Smokers are included (yellow squares). Values are log transformed. The data have been adjusted for the effect of measurement batch by regressing out this variable (see Methods section). Red boxplots represent the PD group, and blue boxplots represent the control group. Boxes: median and interquartile range (IQR); whiskers: 1.5 x IQR from the lower and upper quartiles. Each dot represents one individual. **(a)** CS-normalized CI activity. . **(b)** CS-normalized CII activity. **(c)** CS-normalized CIII activity. 16 samples were excluded due to technical issues with the reduction of decylubiquinone (Supplementary Data 1). **(d)** CS-normalized CIV activity. *: $P = 0.017$ (linear regression model).

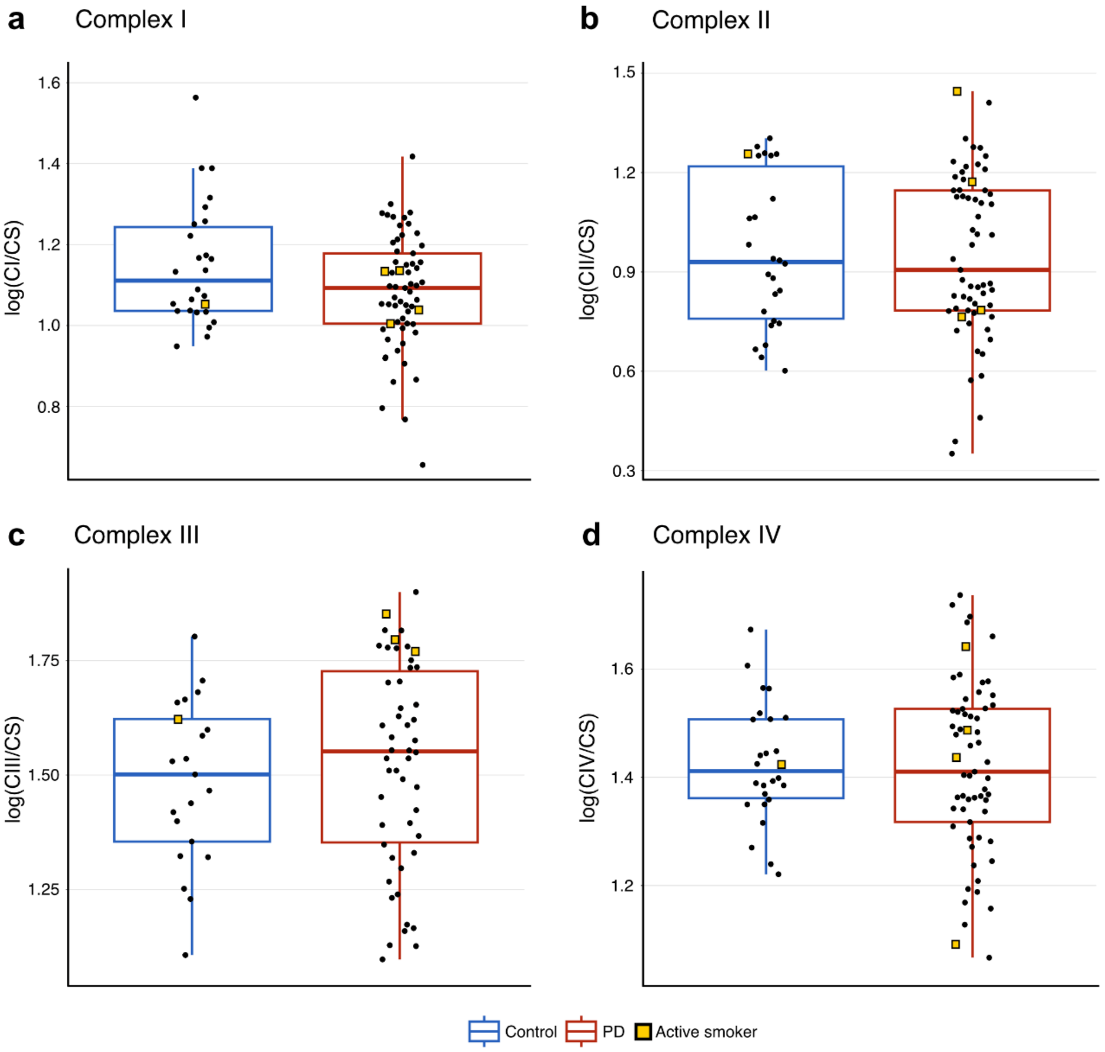

Supplementary Figure 11. Spectrophotometric activity measurement of mitochondrial respiratory complexes in muscle, including smokers and without adjusting for batch effects. Activities of complexes I-IV (CI-CIV), normalized to citrate synthase (CS) activity. Values are log transformed. Smokers (yellow squares) are included. Red boxplots represent the PD group, and blue boxplots represent the control group. Boxes: median and interquartile range (IQR); whiskers: 1.5 x IQR from the lower and upper quartiles. Each dot represents one individual. **(a)** CS-normalized CI activity. **(b)** CS-normalized CII activity. **(c)** CS-normalized CIII activity. 16 samples were excluded from this analysis due to technical issues with the reduction of decylubiquinone (Supplementary Data 1). **(d)** CS-normalized CIV activity.

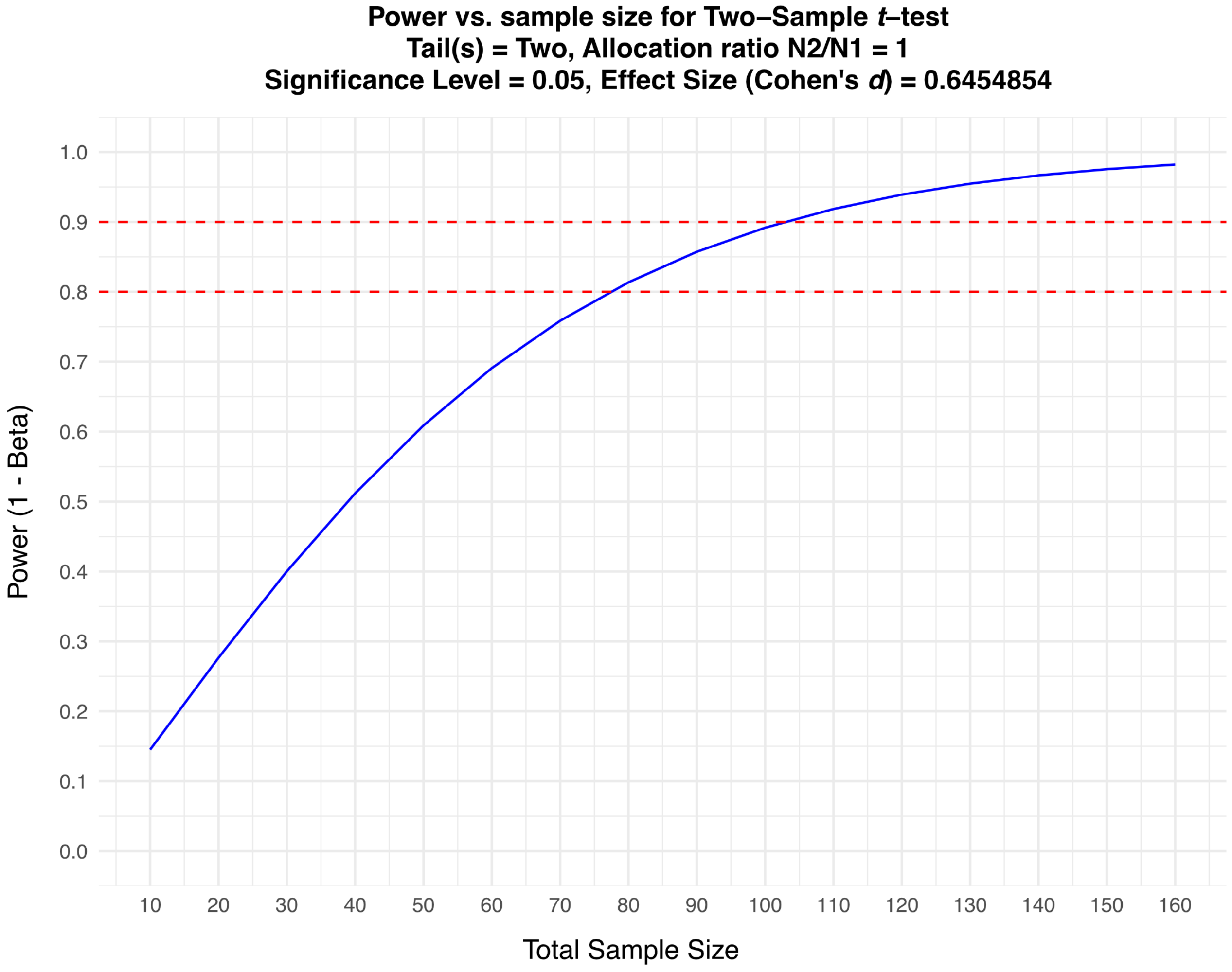

Supplementary Figure 12. Power as a function of sample size for complex I activity data. After adjusting the data for the effect of measurement batch by regressing out this variable (see Methods section), the difference in citrate synthase-normalized complex I activity (CI/CS) between the patient and control groups corresponded to a medium effect size (Cohen's *d* of 0.65). The plot shows the total sample size needed for different power values for a two-sample *t*-test given this effect size. Dashed red lines indicate 80% and 90% power.

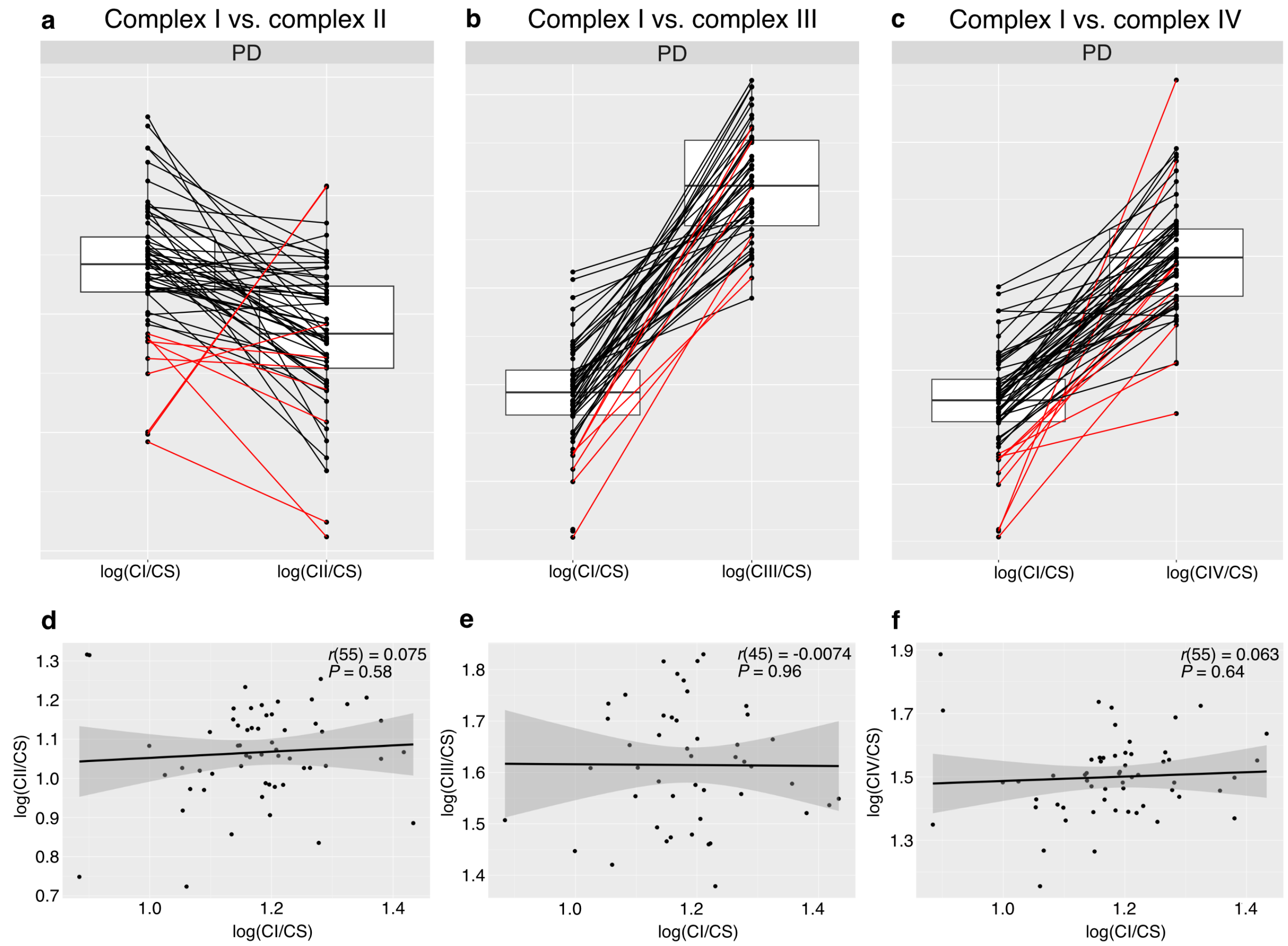

Supplementary Figure 13. Correlation between spectrophotometric activity measurement of CI and CII-IV in individuals with PD

Activities of complexes I-IV (CI-IV), normalized to citrate synthase (CS) activity are shown. Values are log transformed. For the purpose of visualization, the data have been adjusted for the effect of measurement batch by regressing out this variable (see Methods section). Smokers have been removed from the dataset. **(a-c)** Boxplots compare the distributions of individual measurements of CI/CS to the distributions of CII/CS, CIII/CS, and CIV/CS measurements. Boxes: median and interquartile range (IQR); whiskers: 1.5 x IQR from the lower and upper quartiles. Each dot represents one individual. Lines connect corresponding individuals. Red lines indicate individuals with CI/CS activity below the range of controls (the same individuals are indicated in main Figure 3a). **(d-f)** Scatterplots show correlation between CI/CS and CII/CS, CIII/CS, and CIV/CS. Each dot represents one individual.

Supplementary Table 1: Demographic and clinical characteristics per analysis

Method	*n*	Age	Sex (M : F)	Disease duration in years
COX/SDH histochemistry				
PD	68	65.9 (± 7.8)	46 : 22	5.2 (± 4.4)
Control	21	61.1 (± 9.9)	5 : 16	
Quadruple immunohistochemistry				
PD	71	66.2 (± 7.8)	47 : 24	5.2 (± 4.3)
Control	21	61.1 (± 9.9)	5 : 16	
Enzymatic activity (without smokers)				
PD	57	67.1 (± 7.4)	35 : 22	6.5 (± 4.9)
Control	25	65.4 (± 12.0)	8 : 17	
Enzymatic activity (with smokers)				
PD	61	66.8 (± 7.4)	37 : 24	6.5 (± 4.8)
Control	26	65.2 (± 11.8)	8 : 18	
mtDNA analyses (bulk tissue)				
PD (normal CI activity)	8	65.0 (± 6.7)	6 : 2	5.2 (± 1.7)
PD (low CI activity)	9	66.9 (± 10.9)	3 : 6	5.4 (± 2.7)
Control	10	64.9 (± 8.9)	3 : 7	
mtDNA analyses (single fibers)				
PD	6	66.8 (± 6.5)	5 : 1	5.1 (± 4.1)
Control	6	64.8 (± 9.5)	1 : 5	

Age and disease duration are presented as mean ± standard deviation.

Supplementary Table 2: Olympus VS120 fluorescence filters

Filter:	Excitation:	Emission:
DAPI	340-380	430-480
FITC	480-500	507-543
TRITC	543-568	579-631
CY5	630-660	669-741

Supplementary Table 3: Comparison of COX/SDH histochemistry results in the PD and control groups

Variable	Group	Median (IQR)	Test	Test Statistic	*df*	*P*-value
Proportion of COX negative or intermediate muscle fibers per section	PD (*n* = 68)	0 (0.004)	Wilcoxon rank-sum test (two-sided)	*W* = 764	NA	0.584
	Control (*n* = 21)	0 (0.002)				
Number of individuals with any COX negative or intermediate fibers			Pearson's Chi-squared Test (with Yates' continuity correction)	$\chi^2 = 0.07$	1	0.788

	Any negative or intermediate	No negative or intermediate
PD (*n* = 68)	27	41
Control (*n* = 21)	7	14

Abbreviations: COX, cytochrome c oxidase; SDH, succinate dehydrogenase; IQR, interquartile range; *df,* degrees of freedom. Significant *P*-values are in bold. Nominal *P*-values are given.

Supplementary Table 4: Linear mixed effects model of VDAC1 fluorescence intensity level in single muscle fibers

	Dependent variable		
		log(VDAC1)	
Predictors	*B*	95% CI	*P*-value
Disease state (PD)	0.003	-0.068 – 0.074	0.933
Age	1.3e-04	-0.003 – 0.004	0.942
Sex (Male)	0.028	-0.032 – 0.089	0.362
Smoking	-0.099	-0.221 – 0.024	0.115
Batch (Batch 2)[a]	0.188	0.132 – 0.244	**4.9e-11**
Random Effects			
σ^2	0.019		
$\tau_{00\ \text{Subject}}$	0.018		
ICC	0.487		
n_{Subject}	92		
Observations	9073		
Marginal R^2 / Conditional R^2	0.191 / 0.585		

Abbreviations: log(VDAC1), measure of mitochondrial content; *B*, regression coefficient (unstandardized); 95% CI, 95% confidence interval of the regression coefficient; σ^2, residual variance; $\tau_{00\ \text{Subject}}$, random intercept variance; ICC, intraclass correlation coefficient, representing the proportion of total variance in the dependent variable attributable to the grouping structure (i.e, subjects); n_{Subject}, number of study subjects.
Significant *P*-values are in bold. Nominal *P*-values are given.

[a]Immunohistochemistry staining was performed in two batches.

Supplementary Table 5: Linear regression models of complex I and IV level and VDAC1 fluorescence level in large muscle sections areas

	Dependent variable								
	log(NDUFB10/VDAC1)			log(MTCO1/VDAC1)			log(VDAC1)		
Predictors	*B*	95% CI	*P*-value	*B*	95% CI	*P*-value	*B*	95% CI	*P*-value
Disease state (PD)	-0.016	-0.046 – 0.014	0.285	-0.016	-0.056 – 0.023	0.412	-0.016	-0.089 – 0.057	0.668
Age	-0.002	-0.003 – -2.5e-04	**0.022**	-0.002	-0.004 – -3.6e-04	**0.020**	9.0e-05	-0.003 – 0.004	0.959
Sex (Male)	0.017	-0.008 – 0.043	0.182	0.047	0.013 – 0.081	**0.007**	0.010	-0.052 – 0.073	0.746
Smoking	-0.002	-0.054 – 0.050	0.939	-0.007	-0.076 – 0.061	0.829	-0.098	-0.224 – 0.028	0.125
Batch (Batch 2)[a]	-0.099	-0.123 – -0.076	**9.2e-13**	0.052	0.021 – 0.083	**1.4e-03**	0.216	0.159 – 0.274	**6.2e-11**
Observations	92			92			92		
R^2 / R^2 adjusted	0.495 / 0.465			0.190 / 0.143			0.399 / 0.364		

Abbreviations: log(NDUFB10/VDAC1), complex I level adjusted for mitochondrial content; log(MTCO1/VDAC1), complex IV level adjusted for mitochondrial content; log(VDAC1), measure of mitochondrial content; *B*, regression coefficient (unstandardized); 95% CI, 95% confidence interval of the regression coefficient.
Significant *P*-values are in bold. Nominal *P*-values are given.

[a]Immunohistochemistry staining was performed in two batches.

Supplementary Table 6: Linear regression models of enzymatic activity of CI, II, III, IV and citrate synthase in individuals with PD and controls

	log(CI/CS)			log(CII/CS)			log(CIII/CS)			log(CIV/CS)			log(CS)		
Predictors	*B*	95% CI	*P*-value (adjusted)[a]	*B*	95% CI	*P*-value (adjusted)[a]	*B*	95% CI	*P*-value (adjusted)[a]	*B*	95% CI	*P*-value (adjusted)[a]	*B*	95% CI	*P*-value
Disease state (PD)	-0.079	-0.137 – -0.021	**0.008 (0.032)**	-0.043	-0.103 – 0.018	0.163 (0.313)	-0.038	-0.102 – 0.025	0.235 (0.313)	-0.027	-0.089 – 0.035	0.383 (0.383)	0.034	-0.090 – 0.159	0.582
Age	-0.001	-0.004 – 0.003	0.693	0.002	-0.001 – 0.006	0.143	0.008	0.005 – 0.011	**1.3e-05**	0.003	-1.8e-04 – 0.007	0.063	-0.001	-0.008 – 0.006	0.826
Sex (Male)	0.027	-0.027 – 0.082	0.323	0.013	-0.043 – 0.070	0.640	0.006	-0.052 – 0.064	0.846	0.017	-0.041 – 0.076	0.555	-0.023	-0.140 – 0.093	0.690
Batch 1	Reference	-	-	-	-	-	-	-	-	-	-	-	-	-	-
Batch 2	0.067	-0.029 – 0.163	0.171	0.158	0.058 – 0.258	**0.002**	0.095	0.002 – 0.188	**0.045**	0.049	-0.053 – 0.152	0.341	-0.027	-0.233 – 0.180	0.797
Batch 3	-0.125	-0.216 – -0.035	**0.007**	0.115	0.021 – 0.209	**0.017**	-0.239	-0.326 – -0.151	**1.1e-06**	-0.100	-0.197 – -0.004	**0.042**	-0.111	-0.305 – 0.084	0.260
Batch 4	-0.237	-0.321 – -0.153	**3.1e-07**	-0.392	-0.479 – -0.305	**2.1e-13**	-0.307	-0.387 – -0.228	**1.4e-10**	-0.131	-0.221 – -0.042	**0.005**	0.373	0.193 – 0.553	**9.7e-05**
Batch 5	-0.104	-0.191 – -0.018	**0.018**	-0.204	-0.293 – -0.115	**2.1e-05**				-0.195	-0.287 – -0.103	**7.0e-05**	0.182	-0.003 – 0.367	0.053
Batch 6	-0.127	-0.215 – -0.039	**0.005**	-0.324	-0.416 – -0.233	**7.8e-10**	0.089	0.005 – 0.172	**0.038**	-0.018	-0.112 – 0.076	0.703	0.267	0.077 – 0.456	**0.006**
Observations	82			82			67[b]			82			82		
R^2 / R^2 adjusted	0.481 / 0.424			0.777 / 0.752			0.762 / 0.734			0.348 / 0.276			0.363 / 0.293		

Abbreviations: CI, complex I activity; CII, complex II activity; CIII, complex III activity; CIV, complex IV activity; CS, citrate synthase activity; x/CS, activity x (CI, CII, CIII, or CIV) normalized to citrate synthase activity; *B*, regression coefficient (unstandardized); 95% CI, 95% confidence interval of the regression coefficient. Significant *P*-values are in bold. Nominal and adjusted *P*-values are given.

[a]Parentheses show *P*-values adjusted for multiple testing using the Benjamini-Hochberg procedure for four tests, i.e., CI/CS, CII/CS, CIII/CS and CIV/CS between the PD and control groups.
[b]Complex III activity measurements from 15 individuals were excluded from the analysis due to technical issues with the reduction of decylubiquinone.

Supplementary Table 7: Linear regression models of enzymatic activity of CI, II, III, IV and citrate synthase in individuals with PD and controls with smokers included

	log(CI/CS)			log(CII/CS)			log(CIII/CS)			log(CIV/CS)			log(CS)		
Predictors	*B*	95% CI	*P*-value (adjusted)[a]	*B*	95% CI	*P*-value (adjusted)[a]	*B*	95% CI	*P*-value (adjusted)[a]	*B*	95% CI	*P*-value (adjusted)[a]	*B*	95% CI	*P*-value
Disease state (PD)	-0.070	-0.128 – -0.013	**0.017** (0.068)	-0.043	-0.102 – 0.016	0.148 (0.296)	-0.029	-0.091 – 0.033	0.358 (0.388)	-0.026	-0.087 – 0.034	0.388 (0.388)	0.028	-0.098 – 0.153	0.663
Age	-0.001	-0.004 – 0.003	0.727	0.002	-0.001– 0.005	0.183	0.008	0.004 – 0.011	**1.5e-05**	0.003	-2.7e-04 – 0.006	0.071	3.2e-04	-0.007 – 0.007	0.927
Sex (Male)	0.018	-0.036 – 0.071	0.510	0.021	-0.034 – 0.075	0.455	0.008	-0.048 – 0.064	0.785	0.026	-0.030 – 0.082	0.351	-0.047	-0.164 – 0.069	0.420
Smoking	-0.102	-0.214 – 0.009	0.071	0.046	-0.068 – 0.161	0.424	0.126	0.009 – 0.242	**0.035**	-0.044	-0.161 – 0.073	0.453	-0.127	-0.369 – 0.116	0.303
Batch 1	Reference	-	-	-	-	-	-	-	-	-	-	-	-	-	-
Batch 2	0.047	-0.048 – 0.142	0.325	0.163	0.066 – 0.260	**0.001**	0.088	-0.003 – 0.179	0.057	0.057	-0.043 – 0.156	0.259	-0.037	-0.243 – 0.170	0.724
Batch 3	-0.125	-0.216 – -0.034	**0.008**	0.113	0.019 – 0.207	**0.019**	-0.242	-0.329 – -0.155	**6.1e-07**	-0.103	-0.199 – -0.007	**0.036**	-0.099	-0.298 – 0.10	0.325
Batch 4	-0.236	-0.321 – -0.152	**3.8e-07**	-0.393	-0.480 – -0.305	**1.4e-13**	-0.307	-0.386 – -0.229	**8.7e-11**	-0.132	-0.221 – -0.042	**0.004**	0.376	0.191 – 0.561	**1.2e-04**
Batch 5	-0.101	-0.187 – -0.015	**0.022**	-0.212	-0.300 – -0.124	**8.2e-06**	-	-	-	-0.204	-0.294 – -0.113	**2.5e-05**	0.183	-0.005 – 0.370	0.056
Batch 6	-0.114	-0.202 – -0.026	**0.012**	-0.323	-0.414 – -0.223	**4.7e-10**	0.091	0.009 – 0.173	**0.031**	-0.017	-0.109 – 0.075	0.716	0.292	0.100 – 0.483	**0.003**
Observations	87			87			71[b]			87			87		
R^2 / R^2 adjusted	0.447 / 0.382			0.784 / 0.759			0.776 / 0.747			0.379 / 0.306			0.375 / 0.302		

Abbreviations: CI, complex I activity; CII, complex II activity; CIII, complex III activity; CIV, complex IV activity; CS, citrate synthase activity; x/CS, activity x (CI, CII, CIII, or CIV) normalized to citrate synthase activity; *B*, regression coefficient (unstandardized); 95% CI, 95% confidence interval of the regression coefficient. Significant *P*-values are in bold. Nominal and adjusted *P*-values are given.

[a]Parentheses show *P*-values adjusted for multiple testing using the Benjamini-Hochberg procedure for four tests, i.e., CI/CS, CII/CS, CIII/CS and CIV/CS between the PD and control groups.
[b]Complex III activity measurements from 16 individuals were excluded from the analysis due to technical issues with the reduction of decylubiquinone.

Supplementary Table 8: Linear regression model of enzymatic activity of CI in individuals with PD

	Dependent variable		
		log(CI/CS)	
Predictors	*B*	95% CI	*P*-value
Age	0.001	-0.005 – 0.006	0.765
Sex (Male)	0.025	-0.053 – 0.102	0.521
MDS-UPDRS III score	0.002	-0.002 – 0.005	0.317
MoCA score[a]	0.007	-0.006 – 0.019	0.286
Disease duration (months)	-2.1e-04	-8.6e-04 – 4.4e-04	0.520
Batch 1	Reference	-	-
Batch 2	0.035	-0.096 – 0.165	0.594
Batch 3	-0.181	-0.304 – -0.059	**0.005**
Batch 4	-0.273	-0.395 – -0.150	**5.2e-05**
Batch 5	-0.119	-0.236 – -0.002	**0.047**
Batch 6	-0.128	-0.235 – -0.021	**0.020**
Observations	53		
R^2 / R^2 adjusted	0.490 / 0.368		

Abbreviations: CI, complex I activity; CS, citrate synthase activity; CI/CS, complex I activity normalized to citrate synthase activity; *B*, regression coefficient (unstandardized); 95% CI, 95% confidence interval of the regression coefficient; MDS-UPDRS III score, sum of MDS-UPDRS part III; MoCA: Montreal Cognitive Assessment; Disease duration (months), duration of motor symptoms in months.
Significant *P*-values are in bold. Nominal *P*-values are given.

[a]Four individuals were omitted from the analysis due to missing MoCA scores.

Supplementary Table 9. Comparison of clinical and demographic characteristics between the PD subgroups

Variable	CI activity subgroup	Mean ± SD	Median (IQR)	Test	Test Statistic	*df*	*P*-value	Adjusted *P*-value (BH)
Age at onset	Within control range (*n* = 48)	61.5 ± 7.8	61.1 (12.4)	Student's *t*-test (two-sided)	*t* = -1.78	55	0.080	0.200
	Below control range (*n* = 9)	56.0 ± 11.7	54.1 (12.3)					
MoCA score	Within control range[a] (*n* = 44)	24.8 ± 3.2	25 (4)	Student's *t*-test (two-sided)	*t* = 0.38	51	0.703	0.879
	Below control range (*n* = 9)	25.2 ± 3.3	25 (3)					
MDS-UPDRS III score	Within control range (*n* = 48)	27.6 ± 11.0	25.0 (16.3)	Wilcoxon rank-sum test (two-sided)	*W* = 281.5	NA	0.155	0.258
	Below control range (*n* = 9)	21.6 ± 8.7	21.0 (9.0)					
Motor phenotype				Fisher's exact test (two-sided)	NA	NA	1	1
Within control range (*n* = 48)	TD/PIGD: 19/21							
Below control range (*n* = 9)	TD/PIGD: 4/5							
Sex				Fisher's exact test (two-sided)	NA	NA	**0.020**	0.100
Within control range (*n* = 48)	Male/Female: 33/15							
Below control range (*n* = 9)	Male/Female: 2/7							

Abbreviations: CI, complex I; IQR, interquartile range; *df,* degrees of freedom; BH, Bejamini-Hochberg procedure; Age at onset, Age at onset of motor symptoms; MoCA, Montreal Cognitive Assessment; MDS-UPDRS III, International Parkinson and Movement Disorder Society Unified Parkinson's Disease Rating Scale Part III; TD, tremor dominant; PIGD, postural instability/gait difficulty.

Significant *P*-values are in bold. Nominal and adjusted *P*-values are given. Adjusted *P*-values were calculated using the Benjamini-Hochberg procedure to account for multiple comparisons

[a]MoCA scores were not available from four individuals in the PD group with CI activity within control range.

Supplementary Table 10: Linear mixed effects model of complex I level in single muscle fibers in individuals with PD and controls.

The data are restricted to individuals with available complex I activity data.

	Dependent variable		
	log(NDUFB10/VDAC1)		
Predictors	*B*	95% CI	*P*-value
Disease state (PD)	-0.024	-0.061 – 0.014	0.216
Age	-0.001	-0.003 – 0.001	0.180
Sex (Male)	0.016	-0.016 – 0.048	0.325
Batch (Batch 2)[a]	-0.096	-0.130 – -0.062	**3.7e-08**
Random Effects			
σ^2	0.003		
$\tau_{00\ \text{Subject}}$	0.004		
ICC	0.509		
n_{Subject}	62		
Observations	6073		
Marginal R^2 / Conditional R^2	0.253 / 0.634		

Abbreviations: log(NDUFB10/VDAC1), complex I level adjusted for mitochondrial content; *B*, regression coefficient (unstandardized); 95% CI, 95% confidence interval of the regression coefficient; σ2, residual variance; $\tau_{00\ \text{Subject}}$, random intercept variance; ICC, intraclass correlation coefficient, representing the proportion of total variance in the dependent variable attributable to the grouping structure (i.e, subjects); n_{Subject}, number of study subjects.
Significant *P*-values are in bold. Nominal *P*-values are given.

[a]Immunohistochemistry staining was performed in two batches.

Supplementary Table 11. ANOVA comparison evaluating the impact of complex I quantity on complex I activity

Model	Res.Df	RSS	Df	Sum of Sq	*F*	*Pr(>F)*
Model 1	57	0.64	NA	NA	NA	NA
Model 2	58	0.76	-1	-0.12	10.98	**0.002**

Comparison of two linear regression models using ANOVA.
Model 1: log(CI/CS)[a]~Disease state + Age + Sex + log(NDUFB10/VDAC1)[b]
Model 2: log(CI/CS)[a]~Disease state + Age + Sex
The table presents the residual degrees of freedom (Res.Df), residual sum of squares (RSS), the difference in degrees of freedom (Df), the difference in residual sum of squares (Sum of Sq), the *F*-statistic (*F*), and the corresponding *P*-value (*Pr(>F)*). Significant *P*-value in bold. Nominal *P*-value is given.
[a]The data have been adjusted for the effect of measurement batch by regressing out this variable.
[b]The data have been adjusted for the effect of staining batch by regressing out this variable.

Supplementary Table 12. Linear regression model of enzymatic activity of CI in individuals with PD and controls.

The data are restricted to individuals with available complex I immunohistochemistry data.

	Dependent variable		
	log(CI/CS)[a]		
Predictors	*B*	*95% CI*	*P*-value
Disease state (PD)	-0.035	-0.101 – 0.032	0.301
Age	-0.001	-0.004 – 0.003	0.699
Sex (Male)	0.017	-0.042 – 0.076	0.573
log(NDUFB10/VDAC1)[b]	0.806	0.319 – 1.293	**0.002**
Observations	62		
R^2 / R^2 adjusted	0.216 / 0.161		

Abbreviations: CI, complex I activity; CS, citrate synthase activity; CI/CS, complex I activity normalized to citrate synthase activity; *B*, regression coefficient (unstandardized); 95% CI, 95% confidence interval of the regression coefficient; log(NDUFB10/VDAC1), complex I level adjusted for mitochondrial content. Significant *P*-values are in bold. Nominal *P*-values are given.

[a]The data have been adjusted for the effect of measurement batch by regressing out this variable.
[b]The data have been adjusted for the effect of staining batch by regressing out this variable.

Supplementary Table 13: Linear mixed effects model of mtDNA copy number in single muscle fibers

	Dependent variable		
	mtDNA copy number per micro dissected area (μm^2)		
Predictors	*B*	95% CI	*P*-value
Disease state (PD)	1.44	-0.67 – 3.54	0.179
Age	2.2e-03	-0.11 – 0.11	0.968
Sex (Male)	-2.33	-4.60 – -0.06	**0.044**
Plate 1	Reference	-	**-**
Plate 2	-5.33	-7.58 – -3.07	**5.6e-06**
Plate 3	-7.22	-9.38 – -5.07	**3.1e-10**
Plate 4	-8.86	-11.15 – -6.57	**8.6e-13**
Plate 5	-5.40	-8.17 – -2.64	**1.6e-04**
Plate 6	-5.95	-8.70 – -3.20	**3.0e-05**
Plate 7	-6.61	-9.44 – -3.78	**7.1e-06**
Plate 8	-6.56	-9.51 – -3.60	**1.9e-05**
Plate 9	-6.55	-9.22 – -3.88	**2.6e-06**
Plate 10	-5.14	-7.82 – -2.46	**2.1e-04**
Plate 11	-4.44	-7.13 – -1.76	**1.3e-03**
Plate 12	-5.62	-8.32 – -2.92	**5.8e-05**
Random Effects			
σ^2	11.289		
$\tau_{00\ \mathrm{Subject}}$	0.885		
ICC	0.073		
n_{Subject}	12		
Observations	223		
Marginal R^2 / Conditional R^2	0.297 / 0.348		

Abbreviations: *B*, regression coefficient (unstandardized); 95% CI, 95% confidence interval of the regression coefficient; Plate, qPCR plate; σ^2, residual variance; $\tau_{00\ \mathrm{Subject}}$, random intercept variance; ICC, intraclass correlation coefficient, representing the proportion of total variance in the dependent variable attributable to the grouping structure (i.e, subjects); n_{Subject}, number of study subjects.
Significant *P*-values are in bold. Nominal *P*-values are given.

Supplementary Table 14: Linear mixed effects model of mtDNA major arc deletion levels in single muscle fibers

	Dependent variable		
	mtDNA deletion fraction		
Predictors	*B*	95% CI	*P*-value
Disease state (PD)	0.002	-0.058 – 0.062	0.945
Age	2.0e-03	-0.003 – 0.003	0.899
Sex (Male)	-0.001	-0.066 – 0.065	0.983
Plate 1	Reference	-	-
Plate 2	-0.026	-0.053 – 0.001	0.056
Plate 3	-0.015	-0.041 – 0.010	0.237
Plate 4	0.012	-0.015 – 0.040	0.373
Plate 5	-0.008	-0.072 – 0.055	0.794
Plate 6	-0.012	-0.075 – 0.051	0.710
Plate 7	-0.051	-0.115 – 0.013	0.115
Plate 8	-0.019	-0.084 – 0.046	0.559
Plate 9	0.009	-0.053 – 0.070	0.786
Plate 10	0.011	-0.05 1– 0.073	0.721
Plate 11	5.0e-03	-0.061 – 0.062	0.986
Plate 12	0.002	-0.060 – 0.063	0.960
**Random Effects**			
σ^2	0.002		
$\tau_{00\ \mathrm{Subject}}$	0.001		
ICC	0.426		
n_{Subject}	12		
Observations	223		
Marginal R^2 / Conditional R^2	0.094 / 0.479		

Abbreviations: *B*, regression coefficient (unstandardized); 95% CI = 95% confidence interval of the regression coefficient; Plate, qPCR plate; σ2, residual variance; $\tau_{00\ \mathrm{Subject}}$, random intercept variance; ICC, intraclass correlation coefficient, representing the proportion of total variance in the dependent variable attributable to the grouping structure (i.e, subjects); n_{Subject}, number of study subjects.
Significant *P*-values are in bold. Nominal *P*-values are given.

Supplementary Table 15. Linear mixed effects model of heteroplasmic load in single muscle fibers

	Dependent variable					
	Heteroplasmic load (amplicon 1)			Heteroplasmic load (amplicon 2)		
Predictors	*B*	95% CI	*P*-value	*B*	95% CI	*P*-value
Disease state (PD)	-3.77	-17.31 – 9.77	0.583	-9.12	-27.14 – 8.90	0.319
Age	0.18	-0.50 – 0.86	0.599	0.60	-0.30 – 1.50	0.191
Sex (Male)	-3.33	-16.79 – 10.14	0.626	-7.43	-25.35 – 10.48	0.414
log(NDUFB10/VDAC1)	-23.48	-70.43 – 23.47	0.325	23.82	-33.63 – 81.27	0.414
Random Effects						
σ^2	206.42			303.32		
$\tau_{00\ Subject}$	59.99			111.39		
ICC	0.23			0.27		
n Subject	12			12		
Observations	154			154		
Marginal R^2 / Conditional R^2	0.050 / 0.264			0.148 / 0.377		

Abbreviations: heteroplasmic load, the sum of the heteroplasmy levels across the mtDNA region; amplicon 1, rCRS coordinates 600-8,600; amplicon 2, rCRS coordinates 9,100-16,200; log(NDUFB10/VDAC1), complex I level adjusted for mitochondrial content; *B*, regression coefficient (unstandardized); 95% CI = 95% confidence interval of the regression coefficient; σ2, residual variance; $\tau_{00\ Subject}$, random intercept variance; ICC, intraclass correlation coefficient, representing the proportion of total variance in the dependent variable attributable to the grouping structure (i.e, subjects); n Subject, number of study subjects.
Significant *P*-values are in bold. Nominal *P*-values are given.

Supplementary Table 16. Wilcoxon rank sum tests (two-sided) of mtDNA data in bulk muscle tissue

mtDNA copy number per *APP*						
Groups	*n*	Median	IQR	Wilcoxon *W*	*Z*	*P*-value
Control	10	10000	3215	55	0.816	0.438
Low PD	9	7312	3111			
Control	10	10000	3215	38	-0.178	0.894
Normal PD	8	9691	16522			
Low PD	9	7312	3111	27	-0.866	0.413
Normal PD	8	9691	16522			

mtDNA deletion proportion						
Groups	*N*	Median	IQR	Wilcoxon *W*	*Z*	*P*-value
Control	10	0.058	0.084	34	-0.898	0.391
Low PD	9	0.091	0.040			
Control	10	0.058	0.084	43	0.267	0.824
Normal PD	8	0.072	0.066			
Low PD	9	0.091	0.040	46	0.962	0.361
Normal PD	8	0.072	0.066			

Abbreviations: Normal PD, PD samples with CS normalized CI activity similar to controls; Low PD, PD samples with low CI normalized CI activity.
Significant *P*-values are in bold. Nominal *P*-values are given.

Supplementary Table 17. Linear regression model of heteroplasmic load in bulk muscle tissue as a function of CI activity and enzymatic activity measurement batch.

	Dependent variable					
	Heteroplasmic load (amplicon 1)			Heteroplasmic load (amplicon 2)		
Predictors	*B*	95% CI	*P*-value	*B*	95% CI	*P*-value
log(CI/CS)	-2.43	-14.35 – 9.50	0.676	8.08	-7.93 – 24.09	0.305
Batch 1	Reference	-	-	-	-	-
Batch 2	8.80	-0.13 – 17.73	0.053	7.06	-4.93 – 19.04	0.234
Batch 3	3.03	-7.12 – 13.17	0.541	4.90	-8.71 – 18.51	0.461
Batch 4	-0.86	-10.08 – 8.37	0.848	1.93	-10.45 – 14.31	0.748
Batch 5	1.77	-8.55 – 12.09	0.724	13.44	-0.41 – 27.29	0.057
Batch 6	0.20	-9.48 – 9.88	0.966	1.63	-11.37 – 14.62	0.796
Observations	27			27		
R^2 / R^2 adjusted	0.220 / -0.014			0.254 / 0.031		

Abbreviations: heteroplasmic load, the sum of the heteroplasmy levels across the mtDNA region; amplicon 1, rCRS coordinates 600-8,600; amplicon 2, rCRS coordinates 9,100-16,200; CI, complex I activity; CS, citrate synthase activity; CI/CS, complex I activity normalized to citrate synthase activity; Batch, enzymatic activity measurement batch; *B*, regression coefficient (unstandardized); 95% CI, 95% confidence interval of the regression coefficient.
Significant *P*-values are in bold. Nominal *P*-values are given.
