# Supplementary material for: Mitochondrial complex I deficiency occurs in skeletal muscle of a subgroup of individuals with Parkinson’s disease
Source: Commun Med (Lond). 2025 Apr 27;5:141. doi: 10.1038/s43856-025-00817-7 (PMC12034802; doi:10.1038/s43856-025-00817-7)
Supplement: Supplementary file 2 — REPORTING SUMMARY [file 43856_2025_817_MOESM2_ESM.pdf]

Corresponding author(s): Charalampos Tzoulis

Last updated by author(s): Feb 28, 2025

Reporting Summary

Nature Portfolio wishes to improve the reproducibility of the work that we publish. This form provides structure for consistency and transparency in reporting. For further information on Nature Portfolio policies, see our Editorial Policies and the Editorial Policy Checklist.

Statistics

For all statistical analyses, confirm that the following items are present in the figure legend, table legend, main text, or Methods section.

n/a	Confirmed	
☐	☒	The exact sample size (*n*) for each experimental group/condition, given as a discrete number and unit of measurement
☐	☒	A statement on whether measurements were taken from distinct samples or whether the same sample was measured repeatedly
☐	☒	The statistical test(s) used AND whether they are one- or two-sided<br>*Only common tests should be described solely by name; describe more complex techniques in the Methods section.*
☐	☒	A description of all covariates tested
☐	☒	A description of any assumptions or corrections, such as tests of normality and adjustment for multiple comparisons
☐	☒	A full description of the statistical parameters including central tendency (e.g. means) or other basic estimates (e.g. regression coefficient) AND variation (e.g. standard deviation) or associated estimates of uncertainty (e.g. confidence intervals)
☐	☒	For null hypothesis testing, the test statistic (e.g. *F*, *t*, *r*) with confidence intervals, effect sizes, degrees of freedom and *P* value noted<br>*Give P values as exact values whenever suitable.*
☒	☐	For Bayesian analysis, information on the choice of priors and Markov chain Monte Carlo settings
☒	☐	For hierarchical and complex designs, identification of the appropriate level for tests and full reporting of outcomes
☐	☒	Estimates of effect sizes (e.g. Cohen's *d*, Pearson's *r*), indicating how they were calculated

Our web collection on statistics for biologists contains articles on many of the points above.

Software and code

Policy information about availability of computer code

Data collection	Fluorescent images and brightfield images were acquired on a slide scanner (Olympus VS120 S6) using VS-ASW-S6 software. Image processing and quantification of fluorescence intensity were performed in ImageJ2, version 2.3.0/1.53f. qPCR assays were carried out on a StepOnePlus™ Real-Time PCR System (ThermoFisher) using StepOne™ Software Version 2.3.
Data analysis	Analysis of clinical data, demographic data, histochemistry data, immunohistochemistry data, enzymatic activity data and mtDNA data were performed using R version 4.3.0 (R Core Team, 2023) in RStudio 2023.03.1 Build 446 (2009-2023 Posit Software, PBC). For analysis of mtDNA sequencing data, FASTQ sequencing files were trimmed using Trimmomatic v0.39. Raw FASTQ files were assessed using FastQC prior and following trimming. Reads were aligned to the hg38 human genome reference using BWA v0.7.17. Reads mapping to the mitochondrial chromosome were extracted and duplicates filtered out using GATK MarkDuplicates. Calling of variants was carried out using Mutserve v2.0.0-rc13. Haplocheck was used to assess potential contamination. Linear mixed effects regression models were performed using the R-package “lme4” V1.1.35.1. Regression model summary tables were obtained using the tab_model function of the sjPlot package V2.8.16. The R-package “ggplot2” V3.4.4 was used for plots. Adjusting data for the effect of batch was achieved using the “adjust” function of the R-package “datawizard” V0.9.1. Calculation of power and sample size was performed using pwr V1.3.0 and rstatix V0.7.2.

For manuscripts utilizing custom algorithms or software that are central to the research but not yet described in published literature, software must be made available to editors and reviewers. We strongly encourage code deposition in a community repository (e.g. GitHub). See the Nature Portfolio guidelines for submitting code & software for further information.

Data

Policy information about availability of data

All manuscripts must include a data availability statement. This statement should provide the following information, where applicable:

- Accession codes, unique identifiers, or web links for publicly available datasets
- A description of any restrictions on data availability
- For clinical datasets or third party data, please ensure that the statement adheres to our policy

The immunohistochemistry, enzymatic activity and mtDNA qPCR data generated in this study, as well as the source data for Figures 1-5, are provided in the Supplementary Data files available at figshare: https://doi.org/10.6084/m9.figshare.c.7581485.v3. The sequencing data of the bulk tissue samples and the single muscle fiber samples are available in the Federated European Genome-phenome Archive (FEGA) Norway (accession number: EGAD50000000946). Other data are available from the corresponding author on reasonable request.

Research involving human participants, their data, or biological material

Policy information about studies with human participants or human data. See also policy information about sex, gender (identity/presentation), and sexual orientation and race, ethnicity and racism.

Reporting on sex and gender	The study included 83 individuals with Parkinson's disease (PD), of whom 54 were males and 29 were females, as well as 29 neurologically healthy controls, of whom 8 were males and 21 females. This larger proportion of males in the PD group was mainly related to the higher prevalence of PD in males, and the fact that most control individuals were recruited among the spouses of the individuals with PD. Sex was assigned based on self-reporting and was included as a variable in statistical analyses.
Reporting on race, ethnicity, or other socially relevant groupings	Race, ethnicity or other social grouping were not considered.
Population characteristics	The study included 83 individuals with Parkinson's disease (54 males and 29 females, age 66.2 ± 7.4 years) and 29 controls with no known neurodegenerative disease (8 males and 21 females, age 65.4 ± 11.2 years). Clinical and demographic characteristics of the total study population are provided in Table 1, while clinical and demographic characteristics per analysis is provided in Supplementary Table 1.
Recruitment	The study included individuals with PD enrolled in the NADPARK study (n = 25) or the STRAT-PARK cohort (n = 58), as well as neurologically healthy controls enrolled in the STRAT-PARK cohort (n = 23) or the STRAT-COG cohort (n = 6). Inclusion and exclusion criteria for these studies are provided in Supplementary Data 1. Participants in these studies were recruited during routine outpatient follow-up at the Department of Neurology, Haukeland University Hospital, Bergen, Norway (STRAT-PARK and NADPARK), the Department of Neurology and Clinical Neurophysiology, St. Olav's University Hospital, Trondheim, Norway (STRAT-PARK), and the Geriatric Medicine Outpatient Clinic, Haraldsplass Deaconess Hospital, Bergen, Norway (STRAT-COG). Healthy controls were primarily recruited among the spouses of individuals with PD participating in the STRAT-PARK cohort, as well as among spouses of individuals with dementia participating in the STRAT-COG cohort.
Ethics oversight	The study was approved by the Regional Committee for Medical and Health Research Ethics, Western Norway (NADPARK: 2018/597, STRAT-PARK: 74985, STRAT-COG: 216664).

Note that full information on the approval of the study protocol must also be provided in the manuscript.

Field-specific reporting

Please select the one below that is the best fit for your research. If you are not sure, read the appropriate sections before making your selection.

☒ Life sciences ☐ Behavioural & social sciences ☐ Ecological, evolutionary & environmental sciences

For a reference copy of the document with all sections, see nature.com/documents/nr-reporting-summary-flat.pdf

Life sciences study design

All studies must disclose on these points even when the disclosure is negative.

Sample size	No sample size calculation was performed, but the study aimed to include a greater number of individuals than what has previously been reported on.
Data exclusions	One PD sample and one control sample exhibited unusually high values of specific citrate synthase (CS) activity, and were removed from the enzymatic activity dataset as outliers (data available in Supplementary Data File 3). One control individual was removed from the study due to suspicion of mitochondrial disease. Measurements from 15 samples were excluded from the analysis of complex III enzymatic activity due to technical issues with the reduction of decylubiquinone used in the assay.
Replication	Histological staining (immunohistochemistry and histochemistry) was performed once for each biological sample. Enzymatic activity measurements were performed once in technical triplicates for eample. In the mtDNA qPCR assay, single muscle fiber samples were analyzed

once in technical triplicates, while bulk muscle samples were analyzed three times in technical triplicates. mtDNA sequencing was performed once for each singe muscle fiber sample and once for each bulk muscle tissue sample.

Randomization

Subjects were not randomized into experimental groups. Assays were conducted on the maximum number of available samples. However, due to limited sample material, not all assays could be performed for every subject.

Blinding

Investigators were not blinded to the disease status of the subjects during data collection or analysis. However, due to the objective nature of the assays involved, blinding was not considered necessary for this study.

Reporting for specific materials, systems and methods

We require information from authors about some types of materials, experimental systems and methods used in many studies. Here, indicate whether each material, system or method listed is relevant to your study. If you are not sure if a list item applies to your research, read the appropriate section before selecting a response.

Materials & experimental systems

n/a	Involved in the study
☐	☒ Antibodies
☒	☐ Eukaryotic cell lines
☒	☐ Palaeontology and archaeology
☒	☐ Animals and other organisms
☒	☐ Clinical data
☒	☐ Dual use research of concern
☒	☐ Plants

Methods

n/a	Involved in the study
☒	☐ ChIP-seq
☒	☐ Flow cytometry
☒	☐ MRI-based neuroimaging

Antibodies

Antibodies used

Primary antibodies: Anti-VDAC1 antibody (Abcam, #ab14734), anti-NDUFB10 antibody (Abcam, #ab196019), anti-MTCO1 antibody (Invitrogen, #459600), and anti-laminin antibody (Sigma-Aldrich, #L8271). Secondary fluorescent antibodies: Alexa FluorTM 488 anti-mouse IgG2b (Invitrogen, #A-21141), Alexa FluorTM 594 anti-rabbit IgG (Invitrogen, #A-11012), Alexa FluorTM 647 anti-mouse IgG2a (Invitrogen, #A-21241) DyLightTM 405 anti-mouse IgG1 (BioLegend, #409109).

Validation

Anti-VDAC1 antibody (Abcam, #ab14734): manufacturer's website states that the antibody has been tested for immunocytochemistry/immunofluorescence (ICC/IF) application in human samples.
Anti-NDUFB10 antibody (Abcam, #ab196019): manufacturer's website states that the antibody has been tested for immunocytochemistry/immunofluorescence (ICC/IF) application in human samples (paraffin).
Anti-MTCO1 antibody (Invitrogen, #459600): manufacturer's website states that the antibody has been tested for immunohistochemistry (paraffin) application in human samples.
Anti-Laminin antibody (Sigma-Aldrich, #L8271): manufacturer's website states that the antibody has been tested for immunohistochemistry (formalin-fixed, paraffin-embedded sections) application in human samples.

To assess the validity of the quadruple immunohistochemistry assay using the above listed antibodies, it was compared to cytochrome c oxidase/succinate dehydrogenase (COX/SDH) histochemical staining in serial sections from a muscle biopsy of an individual with mitochondrial myopathy caused by the common ~5 kb single mtDNA major arc deletion which exhibited multiple COX negative fibers (Supplementary Fig. 5).

Plants

Seed stocks

Report on the source of all seed stocks or other plant material used. If applicable, state the seed stock centre and catalogue number. If plant specimens were collected from the field, describe the collection location, date and sampling procedures.

Novel plant genotypes

Describe the methods by which all novel plant genotypes were produced. This includes those generated by transgenic approaches, gene editing, chemical/radiation-based mutagenesis and hybridization. For transgenic lines, describe the transformation method, the number of independent lines analyzed and the generation upon which experiments were performed. For gene-edited lines, describe the editor used, the endogenous sequence targeted for editing, the targeting guide RNA sequence (if applicable) and how the editor was applied.

Authentication

Describe any authentication procedures for each seed stock used or novel genotype generated. Describe any experiments used to assess the effect of a mutation and, where applicable, how potential secondary effects (e.g. second site T-DNA insertions, mosiacism, off-target gene editing) were examined.
